# Lipidomic Profiling of Sweetpotato During Different Developmental Stages Using LC-ESI-MS/MS

**DOI:** 10.3390/foods14234109

**Published:** 2025-11-29

**Authors:** Zaisu Li, Rong Zhang, Xia Jiang, Ying Liu, Zhangying Wang

**Affiliations:** 1 Department of Agronomy, College of Coastal Agricultural Sciences, Guangdong Ocean University, Zhanjiang 524088, China; 2 Guangdong Academy of Agricultural Sciences & Key Laboratory of Crop Genetic Improvement of Guangdong Province, Crops Research Institute, Guangzhou 510640, China

**Keywords:** sweetpotato, lipidomics profile, LC-ESI-MS/MS, multivariate analysis, metabolic pathways

## Abstract

Despite the nutritional importance of sweetpotato, systematic studies on its lipid metabolism remain largely unexplored. To address this gap, this study investigates the dynamic changes in lipid composition during the development of sweetpotato storage roots using a comprehensive lipidomics approach. Through LC-ESI-MS/MS analysis of ‘Guangshu 79’ (G79), an orange-fleshed sweetpotato cultivar, across five developmental stages (S1–S5), 612 lipid species were identified, spanning five major classes: glycerolipids (GL, 57.6%), glycerophospholipids (GP, 24.6%), sphingolipids (SP, 13.9%), fatty acids (FA, 3.6%), and prenol lipids (PR, 0.3%). Early developmental phases (S1–S2) were characterized by upregulation of structural phospholipids (PC, PE) and energy-storage triglycerides (TG), supporting active membrane biogenesis and carbon allocation. Mid-development (S3) showed peak TG accumulation (1439.30 nmol/g), while later stages (S4–S5) exhibited sphingolipid-mediated signaling (Cer, HexCer) and membrane stabilization through glycolipids (MGDG, DGDG). KEGG pathway analysis revealed glycerophospholipid metabolism (25.8%) and sphingolipid metabolism (19.3%) as dominant pathways. These findings systematically characterize the lipid composition and dynamic changes during sweetpotato storage root development, providing a valuable resource for future research on lipid metabolism in root crops.

## 1. Introduction

The sweetpotato [*Ipomoea batatas* (L.) Lam.] is a herbaceous, perennial vine that belongs to the Convolvulaceae family and the genus Ipomoea. Sweetpotato holds the position of the seventh most important global food crop. It is extensively cultivated in over 100 countries worldwide, serving as a vital staple food and a significant nutritional resource [1,2]. Furthermore, as a globally important crop, it is highly valued for its nutritional and economic significance, providing essential carbohydrates, dietary fiber, vitamins, and minerals, making it a key staple in many regions [3,4]. China leads the world in both cultivation area and production, with 2022 statistics from the Food and Agriculture Organization showing that China’s total sweetpotato planting area reached 2.1573 million hectares and its production amounted to 46.8287 million tons, accounting for 29.72% of the global planting area and 54.05% of global production. In addition to the contributions of various sweetpotato cultivars to food security and industrial applications such as bioenergy and starch production, the quality and functionality of its storage roots, the primary economic and edible part of the crop, have been extensively studied, Previous research on sweetpotatoes has primarily focused on carbohydrates, antioxidants, proteins, and other functional components, with almost no attention to lipids [5,6]. Therefore, this study was conducted using the ‘Guangshu 79’ (G79) cultivar, which is not only a representative and commercially important orange-fleshed sweetpotato in Guangdong Province, South China, but also was identified in our preliminary screening as a cultivar with superior fresh-eating quality and relatively high lipid content.

Lipids are a diverse group of hydrophobic biomolecules that include major classes such as glycerolipids, phospholipids, sphingolipids, sterols, and glycolipids [7,8]. For clarity, key lipid classes analyzed herein include glycerolipids (e.g., TG), glycerophospholipids (e.g., PC), sphingolipids (e.g., Cer), fatty acyls (free fatty acids, FFA), and prenol lipids (e.g., CoQ), each serving distinct roles in energy storage, membrane structure, or signaling. These functions are particularly relevant to sweetpotato, where storage root development as a sink tissue relies on membrane turnover and energy storage. Evidence from other crops highlights the agronomic importance of lipids. In plants, lipids are essential components of cellular membranes, maintaining membrane integrity, fluidity, and permeability. Beyond structural roles, lipids function as energy storage molecules, primarily in the form of triacylglycerols (TAGs), and act as signaling molecules involved in regulating growth, development, and stress responses [6,9]. Beyond these canonical roles, mounting evidence shows that lipid metabolism can directly shape agronomic traits such as yield, quality and stress resilience across crop species. In oilseed crops such as canola, manipulation of key lipid biosynthetic genes (e.g., DGAT1, WRI1) has been shown to significantly enhance seed oil content and overall oil yield under field conditions [10]. These findings highlight the direct contribution of lipid metabolic flux to agronomic performance in high-oil crops. Similarly, pivotal roles for lipids have been uncovered in non-oil crops. For example, in cotton, balanced sphingolipid and sterol pools are indispensable for cotton fiber elongation; pharmacological or genetic disruption of sphingolipid biosynthesis shortens fibers and hampers secondary-wall deposition [11]. Even rice contain relatively low levels of lipids (2–3% dry weight), yet lipid composition significantly influences cooking and eating quality and post-harvest performance. Higher lipid content in rice grains correlates with lower peak viscosity (PV) during cooking, a key factor affecting rice texture. This lower PV is due to the reduced starch gelatinization and retrogradation, resulting in firmer and less sticky rice, which directly affects consumer preference and overall eating quality [12].

Therefore, to address the lack of systematic lipidomic research in sweetpotato, this study aims to investigate the lipid composition of the Guangshu 79 (G79) cultivar across five developmental stages using liquid chromatography–electrospray ionization tandem mass spectrometry (LC-ESI-MS/MS)-based lipidomic profiling. By characterizing dynamic lipid profile changes during root development, this research seeks to provide insights into lipid metabolism and its potential role in sweetpotato growth and quality formation. The findings will not only deepen our understanding of lipid functions in storage root biology but also support practical strategies for improving the quality, utilization, and breeding of high-value cultivars.

## 2. Materials and Methods

### 2.1. Experimental Materials and Growing Conditions

This experiment used orange-fleshed sweetpotato ‘Guangshu 79’ (G79) as the experimental material. The samples were planted at the Lufeng Experimental Base of the Guangdong Academy of Agricultural Sciences, with planting commencing in May 2023 and transplantation in July 2023. Sweetpotato storage roots were collected as experimental materials at 45 days after transplantation (S1, August 2023), 75 days (S2, September 2023), 105 days (S3, October 2023), 135 days (S4, November 2023), and 165 days (S5, December 2023). A completely randomized block design was employed to randomly select sweetpotatoes of uniform size, with 3 medium-sized sweetpotatoes taken from each period. All samples were rinsed with distilled water, dried, and weighed. Subsequently, they were freeze-dried for 72 h and ground into a fine powder. The powdered samples were passed through an 80-mesh sieve and stored in sealed bags at −20 °C until further analysis.

### 2.2. Chemicals and Reagents

HPLC-grade solvents, including acetonitrile (ACN), methanol (MeOH), isopropanol (IPA), dichloromethane (CH_2_Cl_2_), and methyl tert-butyl ether (MTBE), were sourced from Merck (Darmstadt, Germany). Additionally, HPLC-grade formic acid (FA) and ammonium formate (AmFA) were procured from Sigma-Aldrich (St. Louis, MO, USA). Ultrapure water was generated using a Milli-Q system provided by Millipore (Billerica, MA, USA). Furthermore, lipid standards were obtained either from Sigma-Aldrich or Avanti Polar Lipids (Alabaster, AL, USA). The analysis was performed using an Applied Biosystems 6500 QTRAP mass spectrometer (AB SCIEX, Singapore City, Singapore).

### 2.3. Sample Preparation and Extraction

Prior to lipid extraction, a mixture of internal standards covering major lipid classes was added to each sample for quantitative correction. The list of these standards is provided in Supplementary Table S1. The extraction of lipids was performed utilizing a solvent mixture comprising methanol and methyl tert-butyl ether (MTBE). Specifically, 20 mg aliquot of the dried sample was transferred into a 2 mL centrifuge tube, and 1 mL of a lipid extraction solvent (MTBE:MeOH at a ratio of 3:1) was added, and the mixture was vortexed for 30 min. Following this, 300 μL of ultra-pure water was introduced and vortexed for 1 min, after which the sample was allowed to stand at 4 °C for 10 min. The sample was then centrifuged at 12,000 rpm for 3 min at 4 °C, and 400 μL of the supernatant was transferred to a 1.5 mL centrifuge tube. This was concentrated to complete dryness at 20 °C. Next, 200 μL of a lipid complex solution (ACN:IPA at a ratio of 1:1) was added to redissolve the residue. After vortexing for 3 min, the mixture was centrifuged again at 12,000 rpm for 3 min at 4 °C. Finally, 120 μL of the reconstituted solution was collected for LC-MS/MS analysis.

### 2.4. HPLC Conditions

The sample extracts were subjected to analysis using an LC-ESI-MS/MS system, comprising a UPLC (ExionLC AD) (ExionLC AD, Sciex, Singapore) and an MS (QTRAP^®^ 6500+ System) (QTRAP® 6500+ System, Sciex, Singapore). The UPLC conditions were set as follows: a Thermo Accucore™ C30 column (2.6 μm, 2.1 mm × 100 mm i.d.; Thermo Fisher Scientific, Waltham, MA, USA) (2.6 μm, 2.1 mm × 100 mm i.d.) was used; the solvent system consisted of A (acetonitrile/water, 60/40 *v*/*v*, with 0.1% formic acid and 10 mmol/L ammonium formate) and B (acetonitrile/isopropanol, 10/90 *v*/*v*, with 0.1% formic acid and 10 mmol/L ammonium formate); the gradient program was A/B 80:20 *v*/*v* at 0 min, changing to 70:30 *v*/*v* at 2.0 min, 40:60 *v*/*v* at 4 min, 15:85 *v*/*v* at 9 min, 10:90 *v*/*v* at 14 min, 5:95 *v*/*v* at 15.5 min (maintained until 17.3 min), and then back to 80:20 *v*/*v* at 17.3 min (maintained until 20 min); the flow rate was 0.35 mL/min; the temperature was set at 45 °C; and the injection volume was 2 μL. The effluent was directed to an ESI-triple quadrupole-linear ion trap (QTRAP)-MS for analysis.

### 2.5. ESI-MS/MS Conditions

LIT and triple quadrupole (QQQ) scans were acquired on a triple quadrupole-linear ion trap mass spectrometer (QTRAP), QTRAP^®^ 6500+ LC-MS/MS System, equipped with an ESI Turbo Ion-Spray interface, operating in positive and negative ion mode and controlled by Analyst 1.6.3 software (Sciex). The ESI source operation parameters were as follows: ion source, turbo spray; source temperature 500 °C; ion spray voltage (IS) 5500 V (Positive), −4500 V (Neagtive); Ion source gas 1 (GS1), gas 2 (GS2), curtain gas (CUR) were set at 45, 55, and 35 psi, respectively. Instrument tuning and mass calibration were performed with 10 and 100 μmol/L polypropylene glycol solutions in QQQ and LIT modes, respectively. The QQQ scans were performed as Multiple Reaction Monitoring (MRM) experiments, with the collision gas (nitrogen) pressure set at 5 psi. The Declustering Potential (DP) and Collision Energy (CE) for each individual MRM transition were optimized through further adjustments. A tailored set of MRM transitions was monitored for each elution period, corresponding to the specific metabolites eluted during that particular time frame.

### 2.6. Data Analysis and Statistics

The significance of differences among datasets from various groups was assessed using analysis of variance (ANOVA), with statistical significance set at *p* < 0.05. To further explore these differences, multivariate statistical analyses, specifically principal component analysis (PCA) and orthogonal partial least squares discriminant analysis (OPLS-DA), were conducted using Metware Cloud, a free online platform for data analysis (https://cloud.metware.cn, accessed on 1 April 2025). These analyses were crucial for identifying distinct patterns and variations between the datasets. All analyses were conducted in triplicate to guarantee accuracy, and all mapping data have been standardized.

## 3. Results and Discussion

### 3.1. Lipid Composition of Sweetpotato G79

**Figure 1:**
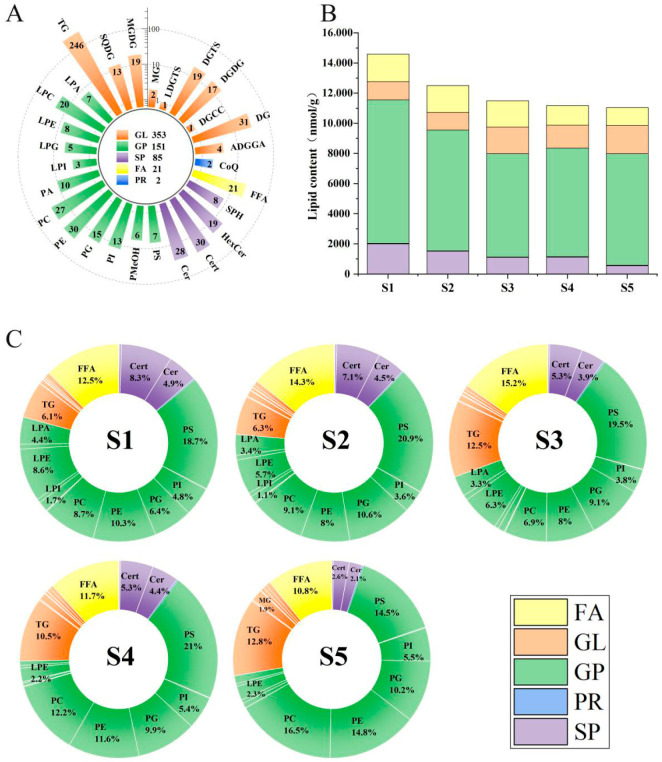
Lipidomic Dynamics Across Five Developmental Stages in Sweetpotato Line G79. (**A**) Number of lipids identified in 28 lipid subclasses and 5 lipid subcategories. (**B**) Stacked plot of total lipid categories across five developmental stages. (**C**) Percentage of subclass lipid content of five developmental stages.

In this study, we conducted a detailed lipidomics analysis of the sweetpotato variety G79, leading to the identification of 612 lipids (comprehensively listed in Table S3). Previous studies have reported the identification of 545 lipids in cassava flour [13,14]. Quantitative analysis revealed that lipids constituted approximately 0.8% of the storage root dry weight across all developmental stages, with total lipid content ranging from 11,037.72 to 14,583.46 nmol/g. These lipids were systematically classified into two hierarchical levels, with primary classification dividing them into five major groups spanning 28 subclasses: fatty acids (FA, 21 species), glycerolipids (GL, 353 species), glycerophospholipids (GP, 151 species), prenol lipids (PR, 2 species), and sphingolipids (SP, 85 species) (Figure 1A). Among these, GL were the most abundant, comprising 57.59% of the total lipids, underlining their significant role in the lipid metabolism of G79 sweetpotatoes [15]. GP followed, accounting for 24.63%, while SP made up 13.87%. In contrast, FA and PR represented smaller fractions, at 3.59% and 0.33%, respectively. This dominance of GL is in line with lipidomic data from other root crops, such as cassava flour [13], reinforcing the view that GL constitute a core component of root lipidomes.

From the secondary classification perspective, the GL was primarily composed of triglycerides (TG, 246 species, 40.2% of total lipids), along with notable quantities of diglycerides (DG, 31 species, 5.1%) and monosaccharide diglycerides (MGDG, 19 species, 3.1%), collectively demonstrating the storage root’s specialized lipid storage system [16]. The glycerophospholipid category (GP, 151 species, 24.7%) featured substantial numbers of phosphatidylethanolamines (PE, 30 species, 4.9%), phosphatidylcholines (PC, 27 species, 4.4%), and phosphatidylglycerols (PG, 15 species, 2.5%), highlighting their essential membrane functions [17]. Sphingolipids (SP, 85 species, 13.9%) included phytoceramides (Cert, 30 species, 4.9%), ceramides (Cer, 28 species, 4.6%), hexosylceramides (HexCer, 19 species, 3.1%), and sphingosine (SPH, 8 species, 1.3%), indicating active signaling networks [18]. Minor components comprised free fatty acids (FA, 21 species, 3.4%) and prenol lipids (PR, 2 species, 0.3%). The striking predominance of TG species (246 out of 612 total lipids) strongly supports their central metabolic role, while the diversity of structural and signaling lipids (totaling 236 species) provides novel insights into storage root development mechanisms, establishing this lipid inventory as a valuable resource for future sweetpotato research.

Upon a thorough comprehension of the characteristics of lipid composition, we proceeded to conduct an in-depth analysis of the dynamic variations in the content of key lipids. Quantitative analysis revealed GP as the most abundant lipid class across all developmental stages (S1–S5), with total content ranging from 6846.84 nmol/g (S3) to 9514.68 nmol/g (S1). GL ranked second, showing a progressive increase from 1166.64 nmol/g (S2) to 1867.41 nmol/g (S5), while FA maintained relatively stable levels (1189.25–1824.42 nmol/g). SP exhibited a declining trend, decreasing from 2004.46 nmol/g (S1) to 552.41 nmol/g (S5), and PR remained the least abundant (27.21–34.05 nmol/g) (Figure 1B, Table 1).

As shown in Figure 1C, the major GP subclasses PC and PE exhibited a characteristic biphasic pattern (initial decrease followed by increase) during storage root development. PC declined from 1274.27 (S1) to 787.70 (S3; 38.2% decrease) before rebounding to 1824.61 (S5), while PE decreased from 1495.08 (S1) to 914.32 (S3; 38.9% decrease) and subsequently increased to 1637.73 (S5). These coordinated changes likely reflect dynamic membrane system remodeling during sweetpotato development [19].

Among GL, the storage lipid TG peaked at 1439.30 during S3, representing a significant 62.8% increase from S1, which aligns with the energy storage demands of storage root bulking [20]. Meanwhile, DG and MG showed sustained upward trends, with DG increasing from 46.86 (S1) to 61.79 (S5; 31.9% increase) and MG rising from 97.04 (S1) to 208.38 (S5; 114.8% increase), indicating continuous activation of the glycerolipid metabolic pathway during storage root development. Particularly noteworthy was the significant accumulation of the plant-specific lipid LDGTS during S3 (5.66; 160.7% increase from S1), suggesting its potential involvement in regulating key biological processes of storage root expansion.

Sphingolipids demonstrated a consistent downward trend throughout development, with Cer decreasing from 719.58 (S1) to 237.06 (S5; 67.1% decrease) and HexCer declining from 56.29 (S1) to 26.11 (S5; 53.6% decrease). These changes may be associated with membrane system reorganization and signaling regulation during later developmental stages [21]. Additionally, the glycolipids MGDG and DGDG showed significant accumulation during maturation (S5), increasing by 101.9% and 55.9%, respectively, from S1 levels, potentially contributing to membrane stability maintenance in mature cells. The sharp decline in LPA after S3 (384.07 at S3 → 80.58 at S4) likely reflects dynamic regulation of phospholipase activity during development [22].

Building on our previous characterization of the G79 sweetpotato line, which provided detailed phenotypic data including storage root length, diameter, and fresh weight [23] (Table 2), we observed that fresh weight surged by 229% from S1 to S2 (12.13 → 39.91 g), coinciding with the peak accumulation of membrane lipids (PC/PE) and initial TG buildup (Figure 1B). Notably, the most rapid diameter expansion (S2–S3: 26.13 → 37.55 mm, +44%) aligned with maximal TG levels (1439.30 nmol/g at S3), suggesting TG-driven cell expansion. Later stages (S4–S5) exhibited slowed fresh weight gain (+6.5%) but sustained diameter growth (+15%), paralleled by sphingolipid (Cer) decline and glycolipid (MGDG/DGDG) accumulation, potentially indicating a shift from growth to maturation.

### 3.2. Multivariate Statistical Analysis of Lipids in G79 Across Different Developmental Stages

**Figure 2:**
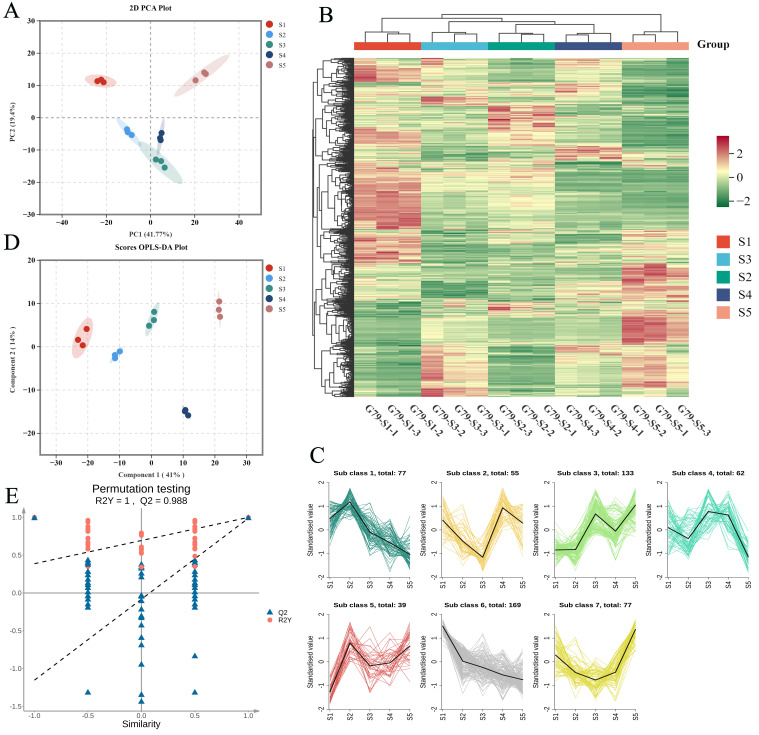
Multivariate statistical analysis of five developmental stages. (**A**) PCA score plot of lipid species. (**B**) Cluster heatmap visualization of lipid metabolites. The red block represents the up-regulated metabolites, the green block represents the down-regulated metabolites. (**C**) K-means cluster analysis of all lipids (**D**) OPLS-DA score plot of lipid species. (**E**) permutation test of the OPLS-DA model.

To comprehensively characterize lipidomic profiles across different developmental stages and identify lipids with differential abundance during development, a multivariate statistical analysis strategy was employed. Initially, unsupervised principal component analysis (PCA) was applied to reveal overall grouping patterns, trends, and potential outliers within the complex dataset. This approach helped elucidate the primary sources of variation among lipid metabolites across different stages [24]. Based on the above approach, we explored lipidomic differences in sweetpotato G79 across five developmental stages (S1, S2, S3, S4, and S5). The PCA score plot (Figure 2A) revealed clear separations among the five groups, with PC1 and PC2 explaining 41.77% and 19.4% of the total variance, respectively. Samples from stage S5 clustered distinctly on the right side of the plot, indicating a unique lipid profile at this stage. Stage S1 samples formed a separate cluster on the upper left. In contrast, samples from stages S2, S3, and S4 clustered closely together near the center of the plot, reflecting relatively similar lipid compositions among these stages. These clustering patterns demonstrate significant lipid profile variations across the developmental stages.

In order to gain a comprehensive understanding of the distribution characteristics of lipid metabolites, a cluster heatmap was subsequently created to depict their relative abundance. The heatmap analysis reveals significant variations in the lipid profiles of sweetpotato across five developmental stages (S1 to S5) (Figure 2B). The clustering results are consistent with the PCA analysis, clearly separating S5 samples as an independent cluster. Stage S1 samples also form a distinct cluster, while samples from stages S2, S3, and S4 group closely together. In the S1 stage, the lipid content is relatively high, as shown by the prominent red regions, suggesting that lipids play a crucial role during early development. In contrast, the S2 stage shows a marked reduction in lipid content, with more green regions, indicating a decrease in lipid accumulation, likely reflecting metabolic shifts or conversion processes. The S3 and S4 stages exhibit relatively stable lipid levels, as indicated by the prevalence of green and yellow regions, which suggests that lipid accumulation becomes more regulated during these stages. In the S5 stage, the most intense red regions are observed, indicating the highest lipid content, which may be associated with energy storage and maturation. Overall, the lipid dynamics across these stages suggest a temporal regulation of lipid metabolism that aligns with the physiological needs and developmental processes of sweetpotato.

K-means cluster analysis was used to analyze the changes in relative lipid content at S1–S5 (Figure 2C). The findings indicated that lipids were organized into seven distinct clusters (Table S4). Among these, clusters 1, 2, and 6 showed the most distinct and biologically relevant patterns during sweetpotato development. Cluster 1, comprising 77 lipids (36.36% GP, 45.45% GL), exhibited an increasing trend peaking at stage S2 followed by a decline. Cluster 2, containing 55 lipids (56.36% GP, 34.55% GL), displayed a biphasic pattern with an initial decrease, a peak at S4, and a subsequent decrease. In contrast, Cluster 6, including 169 lipids, showed a consistent decline throughout development. GP and GL lipids were distributed across multiple clusters, predominantly clusters 1, 2, and 7, while SP and FA were mainly enriched in clusters 1, 4, and 6. These representative clusters highlight the dynamic regulation of lipid metabolism during storage root growth, indicating significant shifts in lipid profiles essential for sweetpotato development.

To further validate these differences, OPLS-DA was applied to enhance group separation. The OPLS-DA score plot (Figure 2D) showed a clear distinction between groups, with Component 1 and Component 2 accounting for 41% and 14% of the variation, respectively. S5 and S1 samples were distinctly separated, while S2, S3, and S4 samples clustered closely together, indicating that lipid profiles from these stages share more similarities. Permutation testing (Figure 2E) confirmed the reliability and robustness of the model, with R^2^Y = 1.0 and Q^2^ = 0.988, indicating strong predictive ability.

### 3.3. Identification and Characterization of Stage-Specific Differential Lipids in G79 Across Developmental Stages

As described above, the OPLS-DA model was applied to differentiate lipid profiles among developmental stages and to calculate the variable importance in projection (VIP) scores for each lipid species. Differentially accumulated lipids (DALs) were then identified by applying the following criteria: VIP ≥ 1, *p*-value < 0.05, and |log_2_ fold change| ≥ 1. The full list of these DALs for all adjacent stage comparisons is available in Supplementary Table S5. After the removal of the same lipid molecules, 334 DLs were identified in sweetpotato storage roots during different developmental stages. The differences among the four developmental stages were mainly attributed to 27 lipid subclasses, covering nearly all major categories except CoQ. GL were the most abundant class, with TG (146 DALs) being the predominant subclass, highlighting their crucial role in energy storage during storage root development. Other significant subclasses included Cert, Cer, LPC and PE.

**Figure 3:**
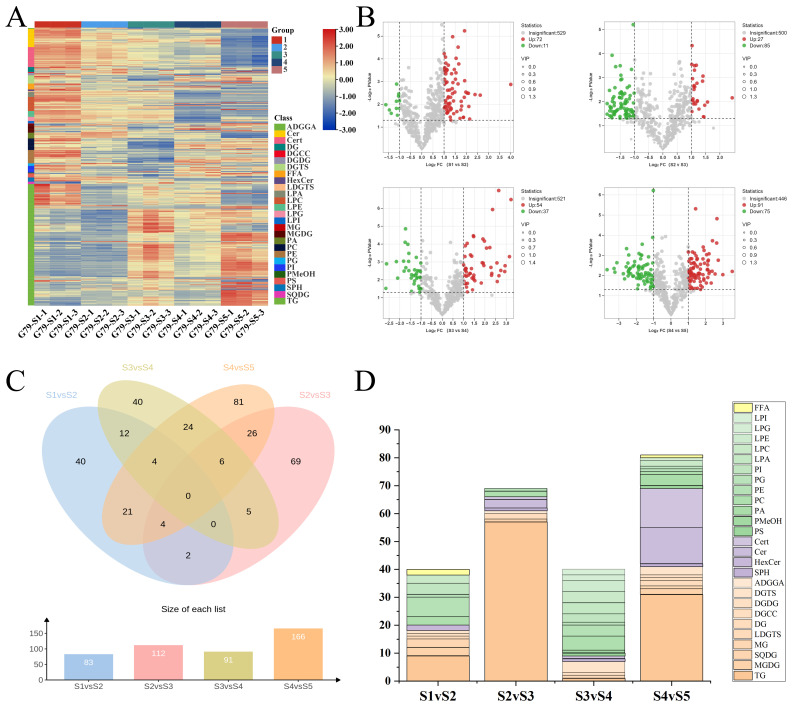
Analysis of lipid differential metabolites in five developmental stages. 10 kinds of sweetpotato. (**A**) Heat map visualization of lipid differential metabolites in G79. (**B**) Volcano plots of S1 vs. S2, S2 vs. S3, S3 vs. S4, S4 vs. S5. (**C**) Venn diagram of differentially accumulated lipids (DALs) across four developmental transitions. (**D**) Stacked bar plot of stage-specific lipid subclasses in differential lipids (DALs).

To assess the differential lipid composition across the five periods, we created a heatmap displaying the lipid subclasses. The heatmap clearly illustrates the dynamic changes in lipid expression at different time points, providing a visual tool to better understand the temporal variations in lipid metabolism and their biological significance (Figure 3A). During S1, TG lipids were predominantly blue, indicating low expression levels. This suggests that fat metabolism or TG synthesis was relatively suppressed or inactive during this period. However, by S5, TG lipids showed a marked increase in expression, represented by red, indicating higher accumulation of TG. This change points to an upregulation of TG synthesis or storage in the later periods, potentially related to energy storage, cell membrane reconstruction, or other biological processes. Overall, the temporal shift in TG lipid expression from low in the first period to high in the fifth demonstrates a dynamic regulation of lipid metabolism, highlighting the active accumulation of TG over time [9,25]. Both Cer and Cert exhibit a clear decrease in expression across the periods. They show high expression in S1 (red), but their levels significantly drop, especially in S5, where they appear blue. This indicates a decrease in these lipid subclasses, potentially reflecting metabolic changes over the course of the experiment [26]. Additionally, PC and PA show a striking change, with their expression abruptly shifting from red (high abundance) to blue (low abundance) at S3. This suggests a significant drop in PC and PA expression at this time point.

Differential lipid expression was assessed by comparing adjacent developmental stages of sweetpotato G79 (S1, S2, S3, S4, and S5). The changes in lipid abundance were visualized using volcano plots (Figure 3B), which effectively illustrate the numbers of significantly up- and down-regulated lipids across developmental transitions. The number of differentially expressed lipids between adjacent stages increased as development progressed. Specifically, in the comparison between S1 and S2, a total of 83 lipids were identified, with 72 upregulated and 11 downregulated. For the S2 vs. S3 comparison, 112 differential lipids were found, with 27 upregulated and 85 downregulated. In the S3 vs. S4 comparison, 91 differential lipids were identified, with 54 upregulated and 37 downregulated. Finally, in the S4 vs. S5 comparison, 166 differential lipids were detected, with 91 upregulated and 75 downregulated. These results highlight the dynamic regulation of lipids, with the increasing number of differential lipids reflecting the more complex lipidomic changes occurring as sweetpotato G79 develops.

To further explore the differential lipids, we constructed a Venn diagram to visually display the overlaps among the four developmental stage comparisons (S1 vs. S2, S2 vs. S3, S3 vs. S4, S4 vs. S5). The analysis indicated that there were no shared differential lipids across all four groups (Figure 3C). This finding highlights the significant stage-specificity of lipid metabolic reprogramming during the development of sweetpotato storage roots, consistent with previous observations of distinct lipid profiles at different developmental stages in other root crops [27]. Then, we discuss the major lipid class alterations observed at each stage, highlighting the dynamic regulation of specific lipid species that are crucial for storage root growth and development. Lipidomic analysis across four adjacent developmental stages of sweetpotato storage roots revealed distinct. The S1 vs. S2 stage featured 40 differential lipids spanning 12 subclasses, with membrane lipids including MGDG, PC, PE, and SQDG species being prominently represented. The S2 vs. S3 stage showed 69 differential lipids with TG subclass comprising the majority (57 species, 83%) accompanied by minor sphingolipid representation. The S3 vs. S4 stage exhibited 40 differential lipids with extensive representation of lysophospholipid subclasses (LPA, LPC, LPE, LPI, LPG accounting for 12 species) and corresponding intact phospholipid species. The S4 vs. S5 stage demonstrated the most complex pattern with 81 differential lipids, notably featuring substantial ceramide representation (27 Cer/Cert species, 33%) and PA species (4 species), highlighting the dynamic shifts in membrane lipid composition during storage root development (Figure 3D) [28].

Our lipidomic analysis revealed distinct stage-specific lipid signatures across four consecutive developmental periods of sweet potato G79 variety storage roots, with no shared differential lipids between adjacent stages, reflecting dynamic lipid metabolic regulation underlying storage root development. During the early developmental stage (S1 vs. S2), significant increases in length (from 9.30 cm to 13.16 cm), diameter (from 15.36 mm to 26.13 mm), and fresh weight (from 12.13 g to 39.91 g) were observed. This stage featured 40 stage-specific differential lipids with significant changes in key membrane lipid components, including downregulation of chloroplast membrane lipids MGDG(16:0_18:2) and MGDG(18:0_18:2), along with a reduction in three SQDG species, changes that may relate to tissue functional transition [29,30]. Meanwhile, upregulation of multiple phospholipids such as PC(18:0_18:1), PC(16:0_18:0), PC(16:0_16:0) and PE species indicated adjustments in membrane phospholipid composition. The S2 vs. S3 transition saw continued growth, with length increasing to 15.97 cm, diameter to 37.55 mm, and fresh weight to 117.03 g. This stage encompassed 69 stage-specific differential lipids, with the most prominent feature being massive downregulation of 56 TG species, indicating major changes in lipid metabolism. This transition also showed upregulation of MGDG(16:0_16:0) and regulation of specific PC species like PC(18:0_18:3) and PC(16:0_16:1), reflecting continued membrane lipid remodeling. The decrease in TG, in parallel with significant fresh weight gain, suggests that lipid remodeling plays a key role in supporting the increased tissue growth and storage root development during this phase. [31]. The S3 vs. S4 transition demonstrated extensive membrane component reorganization through 40 stage-specific differential lipids, with significant upregulation of multiple lysophospholipid species (LPA, LPC, LPE, LPI, LPG) while corresponding intact phospholipids such as PC(16:0_18:3) and PC(18:2_18:3) were downregulated, suggesting enhanced membrane lipid metabolism and remodeling activities [32]. During this period, length increased to 16.48 cm, diameter to 42.03 mm, and fresh weight to 140.76 g. The extensive lipid remodeling likely contributed to the continued growth observed in this stage, supporting membrane stability and the expansion of the storage root. The S4 vs. S5 transition exhibited the most complex lipid change patterns among 81 stage-specific differential lipids, with notable coordinated upregulation of multiple PA species including PA(18:3_16:0), PA(18:2_18:3), PA(18:1_18:3), and PA(16:0_18:2), along with massive accumulation of 27 ceramide species, changes that may relate to membrane stabilization during final storage root maturation [33]. During this stage, the storage root reached its final size, with a length of 16.77 cm, a diameter of 48.38 mm, and a fresh weight of 149.97 g. The increase in ceramides and PA species is likely crucial for membrane integrity and stabilization, which supports the continued maturation and weight gain observed during this period.

Overall, these stage-specific lipid changes demonstrate dynamic regulation of membrane lipid composition during sweet potato storage root development, progressing from a reduction in chloroplast membrane lipids, metabolic regulation of storage lipids, phospholipid remodeling, to final membrane stabilization, constituting a complete developmental regulatory sequence [34]. The results demonstrate that stage-specific lipid changes are closely associated with phenotypic transitions: the degradation of chloroplast lipids coincides with the initiation of root swelling; dramatic metabolic changes in triacylglycerols during the rapid growth phase correspond with accelerated increases in root diameter and fresh weight; while membrane lipid remodeling and ceramide accumulation in mid-late stages are intimately linked to root maturation. These findings reveal the crucial role of lipid metabolic reprogramming in regulating sweet potato storage root, providing new theoretical foundations for quality improvement.

### 3.4. Lipid Metabolism Pathways Analysis

**Figure 4:**
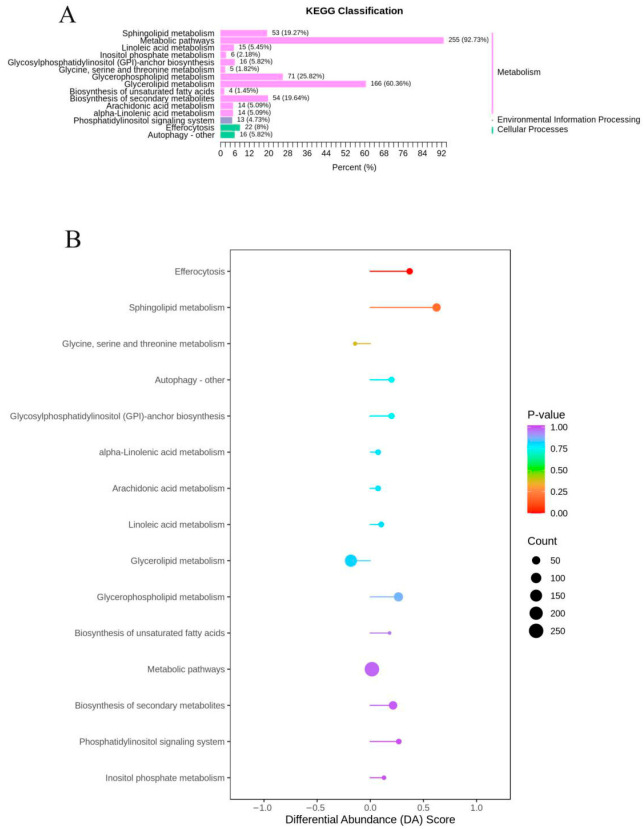
KEGG pathway analysis of lipid differential metabolites and correlation of lipid metabolites with sweetpotato quality characteristics. (**A**) Number of lipids involved in matching pathways. (**B**) Metabolomics view of important metabolic pathways for lipid differential expression. The x-axis represents pathway effects and the y-axis represents pathway enrichment.

A total of 334 identified differential lipids (DLs) were mapped to the KEGG database to gather the associated information and offer a more detailed elucidation of lipid changes within metabolic pathways. These diverse lipids are predominantly involved in 15 metabolic pathways, as depicted in Figure 4A, including biosynthesis of unsaturated fatty acids, sphingolipid metabolism, arachidonic acid metabolism, alpha-linolenic acid metabolism, and sphingolipid metabolism. This suggests that a multitude of biochemical reactions persist throughout the distinct developmental stages of G79. Within these metabolic pathways, 255 significantly differing lipid species are implicated. Metabolic pathways constitute a sequence of biochemical reactions that are indispensable for life, encompassing a diverse array of substances and involving multiple sequential stages.

In this study, lipidomic profiling of sweetpotato G79 during five developmental stages was performed, and KEGG pathway analysis was used to identify key metabolic pathways involved in lipid transformation. The differential abundance (DA) scores and KEGG classification revealed several crucial lipid metabolism pathways.

First, sphingolipid metabolism was one of the most prominent pathways, accounting for 19.27% of the total lipid changes. The metabolism of sphingolipids, such as sphingosine and ceramide, plays a critical role in cellular signaling, stress response, and membrane stability. The significant changes in sphingolipid profiles suggest their involvement in the regulation of membrane function and stress adaptation during the late stages of sweetpotato development. This developmental decrease contrasts with the accumulation of ceramides often associated with stress-induced senescence [26]. The observed decline is consistent with a model in which ceramide-mediated signaling is downregulated as part of the normal maturation program in storage roots. Next, glycerophospholipid metabolism accounted for 25.82% of the lipid changes. This pathway involves key phospholipids such as phosphatidylcholine (PC), phosphatidylethanolamine (PE), and phosphatidylinositol (PI), which are essential for maintaining membrane integrity and fluidity. The alterations in these lipids reflect their important roles in membrane formation, signal transduction, and cellular functions during sweetpotato development. Glycerolipid metabolism, which represented 60.36% of the lipid changes, also played a significant role. This pathway involves triglycerides (TG) and diglycerides (DG). The accumulation of triglycerides in the early stages of development suggests their role as an energy reserve, while the conversion of triglycerides to diglycerides and free fatty acids (FFA) in later stages indicates lipid mobilization during maturation [35].

Additionally, metabolism of unsaturated fatty acids, including linoleic acid and alpha-linolenic acid, contributed 5.09% of the lipid changes. These fatty acids are important components of membrane lipids and play crucial roles in plant stress responses. The significant changes observed in their metabolism highlight their role in maintaining membrane fluidity and modulating stress responses during sweetpotato development. Other notable metabolic pathways involved in lipid transformation included arachidonic acid metabolism, glycosylphosphatidylinositol (GPI)-anchor biosynthesis, and inositol phosphate metabolism, which together contributed 13.63% of the lipid changes. These pathways are involved in cellular processes such as signal transduction, cell growth, and membrane stability [36].

The KEGG analysis and the bubble chart further confirmed the dominance of sphingolipid metabolism and glycerophospholipid metabolism, which showed the largest bubbles and the deepest colors, indicating their higher enrichment and significance in lipid regulation. These findings suggest that lipid metabolism plays a critical role in energy storage, membrane function, and stress adaptation throughout the growth and maturation of sweetpotato G79 [37,38].

## 4. Conclusions

This comprehensive lipidomic investigation of sweetpotato cultivar Guangshu 79 (G79) across five developmental stages (S1–S5) using LC-ESI-MS/MS technology provides valuable insights into the dynamic profiling of lipid metabolism during storage root formation and maturation. A total of 612 distinct lipid species spanning five major classes and 28 subclasses were successfully identified and 334 differentially accumulated lipids were identified covering 27 lipid subclasses, with complete stage specificity demonstrated by Venn diagram analysis showing no shared differential lipids across all four comparison groups. The lipidome underwent a coordinated reprogramming: membrane phospholipids (PC, PE) displayed a biphasic pattern of initial decline and subsequent recovery; storage triglycerides (TG) peaked at 1439.30 nmol/g during the S3 bulking phase; while signaling sphingolipids (Cer, HexCer) showed a consistent downregulation throughout development, marking the transition from cell division to stabilization. KEGG analysis established glycerolipid, glycerophospholipid, and sphingolipid metabolism as the dominant regulatory networks underlying storage root development. These findings not only provide molecular evidence for the temporal regulation governing sweetpotato storage root development but also establish a foundational lipidomic framework for future research. The stage-specific lipid biomarkers, particularly those associated with key metabolic pathways, offer promising targets for future studies. Integrating this lipidomic dataset with transcriptomic analyses will be crucial to elucidate the key regulatory genes controlling these metabolic shifts, ultimately paving the way for genetic improvement of storage root quality.

## Figures and Tables

**Table 1:** Lipid Content (nmol/g) at Five Developmental Stages of Sweetpotato.

Lipids	S1	S2	S3	S4	S5
Sphingosine (SPH)	11.98 ± 1.12 b	14.98 ± 0.65 a	9.49 ± 0.53 c	11.61 ± 0.57 b	7.75 ± 0.34 d
Hexosylceramide (HexCer)	56.29 ± 9.21 a	38.59 ± 4.7 b	29.17 ± 4.02 bc	25.37 ± 5.73 c	26.11 ± 2.84 c
Phytoceramide (Cert)	1216.61 ± 53.24 a	890.62 ± 90.95 b	614.24 ± 90.5 c	594.03 ± 79.22 c	281.5 ± 47.84 d
Ceramide (Cer)	719.58 ± 38.89 a	566.83 ± 67.15 b	444.94 ± 79.36 c	487.18 ± 65.09 bc	237.06 ± 36.07 d
Coenzyme Q (CoQ)	33.83 ± 0.54 a	29.7 ± 1.16 b	34.05 ± 1.51 a	33.68 ± 1.1 a	27.21 ± 0.6 c
Phosphatidylserine (PS)	2728.87 ± 461.76 a	2613.84 ± 103.78 a	2237.67 ± 550.92 ab	2352.6 ± 341.32 ab	1595 ± 389.82 b
Phosphatidylmethanol (PMeOH)	9.94 ± 0.13 bc	8.38 ± 0.29 c	8.34 ± 0.31 c	10.89 ± 1.25 b	14.17 ± 1.49 a
Phosphatidylinositol (PI)	692.95 ± 41.72 a	454.85 ± 18.57 c	434.99 ± 25.97 c	603.01 ± 50.94 b	601.71 ± 36.8 b
Phosphatidylglycerol (PG)	934.52 ± 33.44 c	1319.98 ± 92.31 a	1047.75 ± 53.87 b	1101.32 ± 68.28 b	1120.93 ± 33.59 b
Phosphatidylethanolamine (PE)	1495.08 ± 72.29 a	1000.44 ± 109.46 c	914.32 ± 125.76 c	1297.8 ± 51.36 b	1637.73 ± 71.56 a
Phosphatidylcholine (PC)	1274.27 ± 56.27 bc	1136.51 ± 61.78 c	787.7 ± 100.06 d	1363.59 ± 90.22 b	1824.61 ± 81.63 a
Phosphatidic acid (PA)	17.39 ± 1.22 b	21.33 ± 0.97 a	15.3 ± 1.53 c	13.35 ± 0.69 d	3.45 ± 0.26 e
Lysophosphatidylinositol (LPI)	243.2 ± 6.98 a	140.67 ± 9.92 b	129.76 ± 9.25 b	54.27 ± 0.9 d	89.8 ± 10 c
Lysophosphatidylglycerol (LPG)	95.42 ± 2.77 a	79.5 ± 12.37 b	71.99 ± 5.88 b	26.23 ± 0.17 c	67.33 ± 9.74 b
Lysophosphatidylethanolamine (LPE)	1257.76 ± 42.9 a	716.13 ± 39.92 b	719.35 ± 18.08 b	246.89 ± 12.73 c	254.87 ± 14.33 c
Lysophophatidylcholine (LPC)	118.11 ± 3.22 a	96.41 ± 0.17 b	95.61 ± 3.17 b	49.91 ± 3.4 c	48.96 ± 2.1 c
Lysophosphatidic acid (LPA)	647.17 ± 17.78 a	426.26 ± 16.49 b	384.07 ± 26.75 c	80.58 ± 1.07 e	142.86 ± 10.72 d
Triacylglycerol (TG)	883.79 ± 140.7 b	792.15 ± 59.16 b	1439.3 ± 181.8 a	1173.57 ± 120.99 a	1409.97 ± 181.33 a
Sulfoquinovosyl diacylglycerol (SQDG)	3.54 ± 0.34 c	4.65 ± 0.27 ab	4.05 ± 0.5 bc	3.89 ± 0.47 bc	5.03 ± 0.82 a
Monogalactosyldiacylglycerol (MGDG)	9.33 ± 0.65 c	16.09 ± 2.57 ab	13.78 ± 0.66 b	16.41 ± 1.68 ab	18.85 ± 1.35 a
Monoacylglycerol (MG)	97.04 ± 6.99 c	123.51 ± 23.94 b	112.71 ± 11.55 bc	131.73 ± 8.21 b	208.38 ± 9.58 a
Trimethylhomoserine (LDGTS)	2.17 ± 0.08 c	3.29 ± 0.38 b	5.66 ± 0.54 a	1.13 ± 0.06 d	1.14 ± 0.05 d
Trimethylhomoserine (DGTS)	11.35 ± 0.67 ab	10.44 ± 0.86 bc	8.75 ± 1.21 c	11.86 ± 1.03 ab	13.49 ± 2.23 a
Digalactosyldiacylglycerol (DGDG)	38.71 ± 4.28 c	57.92 ± 3.79 ab	50.25 ± 9.88 abc	46.37 ± 6.43 bc	60.35 ± 4.57 a
Diacylglyceryl-3-0-carboxyhydroxymethylcholine (DGCC)	8.3 ± 0.66 b	11.67 ± 2.91 a	8.64 ± 0.57 b	7.38 ± 0.71 b	3.56 ± 0.24 c
Diacylglycerol (DG)	46.86 ± 4.18 b	53.49 ± 8.61 ab	59.11 ± 11.46 ab	64.17 ± 4 a	61.79 ± 5.31 a
Acyl diacylglyceryl glucuronide (ADGGA)	104.96 ± 14.97 a	93.42 ± 9.54 a	67.89 ± 14.38 b	66.74 ± 8.97 b	84.84 ± 8.05 ab
Free fatty acid (FFA)	1824.42 ± 106.28 a	1784.99 ± 154.44 a	1745.47 ± 114.18 a	1302.86 ± 151.13 b	1189.25 ± 156.37 b

Note: Values are presented as mean ± SD (*n* = 3). Different lowercase letters in the same row indicate significant differences (*p* < 0.05) among development stages.

**Table 2:** Phenotypic Investigation of Sweetpotato at Five Developmental Stages.

Stage	Length (cm)	Diameter (mm)	Fresh Weight (g)
S1	9.30 ± 1.3 a	15.36 ± 0.13 a	12.13 ± 0.06 a
S2	13.16 ± 0.55 b	26.13 ± 0.53 b	39.91 ± 0.80 b
S3	15.97 ± 0.53 c	37.55 ± 1.34 c	117.03 ± 1.52 c
S4	16.48 ± 0.13 c	42.03 ± 0.56 d	140.76 ± 1.45 d
S5	16.77 ± 1.03 c	48.38 ± 0.77 e	149.97 ± 1.67 e

Notes: Values are presented as mean ± SD (*n* = 3). Different lowercase letters in the same column indicate significant differences (*p* < 0.05) among developmental stages.

## Data Availability

The original contributions presented in this study are included in the article/Supplementary Material. Further inquiries can be directed to the corresponding authors.

## Supplementary Materials

The following supporting information can be downloaded at: https://www.mdpi.com/article/10.3390/foods14234109/s1. Table S1: List of internal standards used in lipidomic analysis, Table S2: MRM parameters for 30 representative lipid species, Table S3: Relative Abundance of All Identified Lipids, Table S4: Lipids Assigned to Cluster7 by K-Means Analysis, Table S5: Differential Metabolite Screening Results.
